# Simulated and Experimental Investigation of Mechanical Properties for Improving Isotropic Fracture Strength of 3D-Printed Capsules

**DOI:** 10.3390/ma14164677

**Published:** 2021-08-19

**Authors:** Taeuk Lim, Hao Cheng, Wonil Song, Jasung Lee, Sunghoon Kim, Wonsuk Jung

**Affiliations:** 1 School of Mechanical Engineering, Chungnam National University, Daejeon 34134, Korea; taewook9409@g.cnu.ac.kr (T.L.); chenghao8@naver.com (H.C.); 2 Department of Electronics Convergence Engineering, Wonkwang University, Iksan 54538, Korea; wonil3401@wku.ac.kr (W.S.); grdltkd4@wku.ac.kr (J.L.)

**Keywords:** 3D-printed capsule, mechanical property, isotropic fracture strength, compression experiment

## Abstract

Three-dimensional (3D) printer-based self-healing capsules, embedded in cement composites, were proposed to heal cracks, as they allow for various structural designs of capsules, repeatable fabrication, and strength analysis. Out of many 3D printing methods, such as fusion deposition modeling (FDM), powder layer fusion, and PolyJet printing, FDM was used to design, analyze, and produce new self-healing capsules, which are widely used due to their high-speed, low-cost, and precise manufacturing. However, the PLA extruded in the FDM had low adhesion energy between stacked layers, which caused a degradation of the performance of the self-healing capsule, because it had different strengths depending on the angle between the stacked layers and the applied load within the concrete structure. Therefore, in this paper, specimens were produced, in accordance with ASTM specifications, using the FDM PLA method, and mechanical properties were obtained through tensile, shear, and compression tests. Additionally, the isotropic fracture characteristics of the four types of capsules were analyzed through finite element method analysis. Subsequently, the 3D-printed capsules were produced, and the fracture strength was analyzed in the x, y and z directions of the applied load through a compression test. As a result, the newly proposed capsule design was verified to have an isotropic fracture strength value of 1400% in all directions compared to conventional spherical thin film capsules

## 1. Introduction

There has been increased research interest in the use of self-healing capsules to enhance the safety of concrete structures in terms issues related to cracks [1,2,3,4]. Previously, glass capsules [5,6] and glass cylinders [7,8,9,10] were widely used to encapsulate healing agents [11,12,13], but the disadvantage is that additional structures are needed for protection because glass capsules cannot withstand the concrete mixing process in the form of a cement paste or a metallic wire [14]. To solve this problem, capsule fabrication of various materials, based on substances such as gelatin [15], urea-formaldehyde resin [16,17], paraffin [18,19], silica gel [20,21], polyurethane [22], and expanded clay [23,24], was studied. However, most capsules rely on chemical manufacturing methods or flow control processes [25]. For these manufacturing methods, the capsules were significantly less reproducible. In the case of capsules manufactured using the flow control process, when the size is greater than the order of micrometers, the buoyancy makes it difficult to precisely manufacture the capsules [26].

To compensate for this problem, three-dimensional (3D) printer-based self-healing capsules were proposed that allow for various capsule structural designs, repeatable fabrication, and strength analysis. This 3D printing has become a technology that offers many opportunities in various research fields such as construction, and mechanical and biomedical engineering [27,28,29,30,31,32]. This polymer-based 3D printing technology is well known for obtaining fast and relatively accurate models of high strength; moreover, the method is inexpensive and capable of using complex geometrical shapes [33,34]. Of the many 3D printing methods, such as powder layer fusion, fusion deposition modeling (FDM), and PolyJet printing, FDM methods are widely used due to their high-speed, low-cost, and precise manufacturing [35,36,37,38].

FDM methods can be used to prepare self-healing capsules. The self-healing fluid, located in the capsule, is released through capillary action after the capsule breaks due to external pressure. The spilled healing solution has the ability to react with a chemical catalyst within the solution and start healing cracked concrete [39]. It is preferred that these self-healing capsules have uniform strength in all load directions to achieve uniform fracture properties. However, objects manufactured by the FDM method have different strengths depending on several parameters such as infill density, infill patterns, extrusion temperature, layer thickness, nozzle diameter, and the angle between the stacked printing layers and the applied load [40]. Among the various factors, as the angle is the most important factor in terms of influence [40], the filaments of FDM can resist the load when oriented in the load direction, while filaments oriented in the transverse direction have only the lower bonding forces between them to resist the load [40,41,42,43].

However, no studies have yet been conducted on the application of 3D printer-based capsules to the self-healing field and their isotropic fracture strength. Therefore, finite element method (FEM) analysis should precede the design and fabrication of capsules with isotropic fracture strength. There are related FEM analysis studies such as the FEM analysis of thin-walled composite elements under axial compression [44], and the non-linear analysis of material properties of concrete, such as quasi-plastic behavior [45].

In this study, a new self-healing capsule structure based on 3D printing technology is proposed using the FDM method to overcome the disadvantages of the conventional capsule structure not being isotropic. Therefore, the main research objective is to produce a new structural design of the self-healing capsule with an isotropic fracture strength, and to verify this design with FEM analysis and experiments. First, specimens were produced to obtain the mechanical properties of the FDM specimens through tensile, shear, and compression tests, respectively, for accurate simulation analysis. In particular, the fracture strength according to the angle of the stacked layer and the applied load was verified by structural simulation and compression experiments in which 3D printed capsules had isotropic strength.

## 2. Materials and Methods

**Figure 1:**
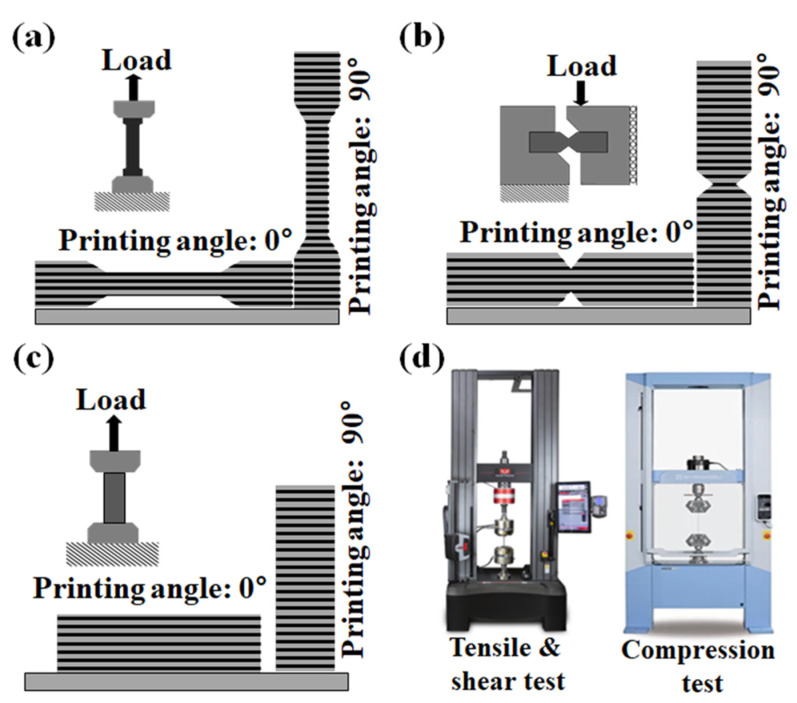
Concept figures of (**a**) tensile test, (**b**) shear test, (**c**) compression test of the specimen produced using the FDM 3D printing method. Material test machine for (**d**) tensile and shear tests (D638), and compression test (D5379).

**Figure 2:**
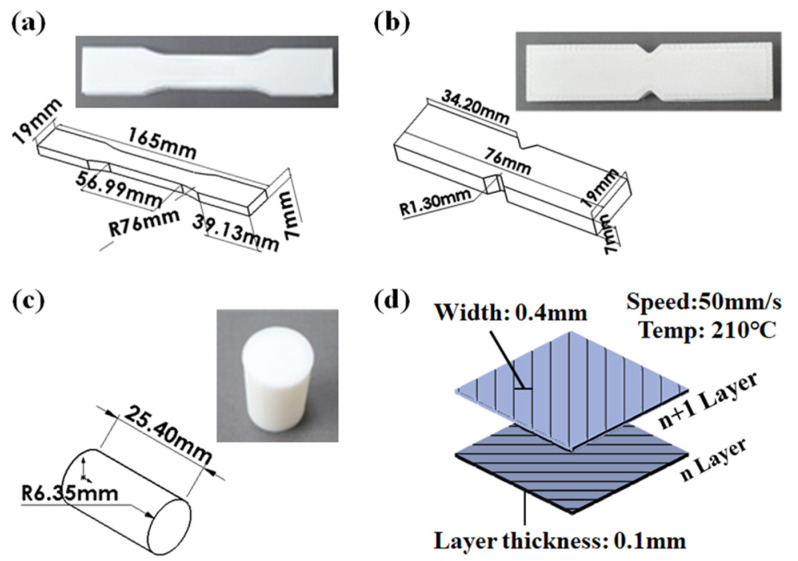
Specimen figures and actual photographs according to ASTM specifications of (**a**) tensile, (**b**) shear, (**c**) compression tests. (**d**) Conditions of the FDM 3D printing method and infill pattern.

**Figure 3:**
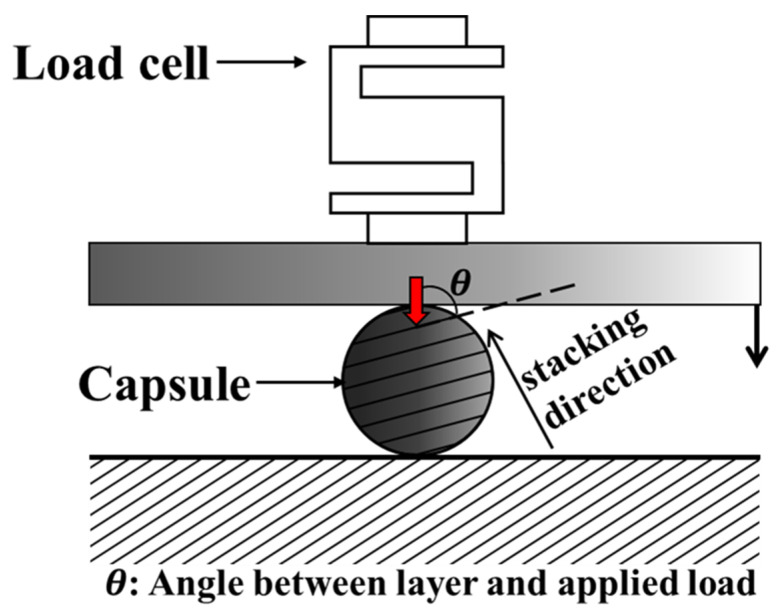
Schematic of FDM-based capsule compression test and definition of angle (theta).

To analyze the applied load and fracture strength of the capsule made by the FDM method, the mechanical properties of the FDM specimen according to the load direction must be obtained. Therefore, a material test was conducted, as shown in Figure 1. Specimens for testing were printed at angles of 0° and 90°, as shown in Figure 1a–c and Figure 2, in accordance with the test proposed in an existing paper [46,47]. This printing angle is defined as the angle between directions of the stacked layer in the FDM method and the applied load, as shown in Figure 3. Tensile, shear, and compression tests were carried out on three specimens for each specimen condition using a material test machine (5982 model, Instron, Norwood, MA, USA and AG-Xplus 100 kN model, Shimadzu, Kyoto, Japan), as shown in Figure 1d. The tensile, shear and compression tests were performed with a constant crosshead speed of 5 mm/min, 2 mm/min, and 1.3 mm/min according to ASTM-D638, D5379, and D695. Detailed specifications and photographs of specimens produced by FDM 3D printers (BRULE, Seoul, Korea), according to ASTM-D638, D5379, and D695, are shown in Figure 2a–c. The specimen was printed at a temperature of 210 °C using a nozzle with a diameter of 0.4 mm, as shown in Figure 2d. The thickness of one layer of the specimen was 0.1 mm; the specimen was printed using PolyLactic Acid (PLA) at a speed of 50 mm/s in a line pattern using Ultimaker2+. PLA is composed of PolyLactic Acid, N, N’-Ethylene Bistearamide, typical antioxidant, and typical pigment material, and the content ratio of each component is the same as shown in Table A1.

## 3. Results

### 3.1. Material Tests of 3D-Printed Specimen

The vulnerable areas of each specimen were investigated by analyzing the fracture location and strength of the FDM-based PLA specimens. Additionally, the mechanical properties of the FDM-based PLA samples were obtained, and the fracture strength of each capsule was analyzed by means of static analysis using the ANSYS program.

**Figure 4:**
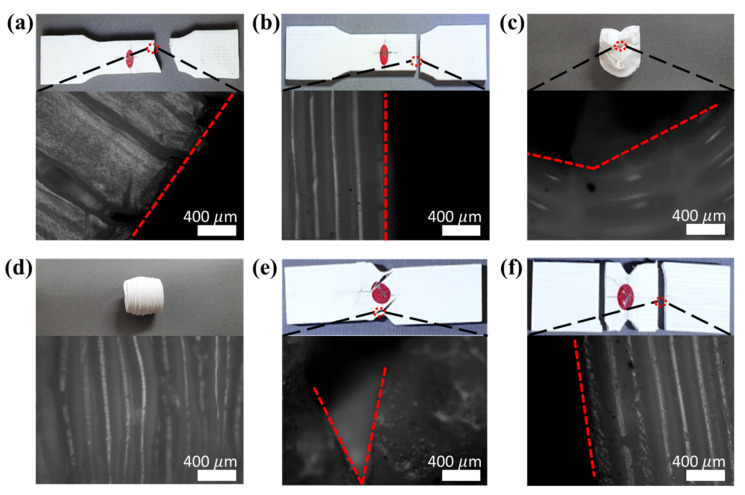
Actual photographs and microscope images of fractured specimens of of (**a**) 0° tensile, (**b**) 90° tensile, (**c**) 0° compression, (**d**) 90° compression, (**e**) 0° shear, and (**f**) 90° shear tests.

**Figure 5:**
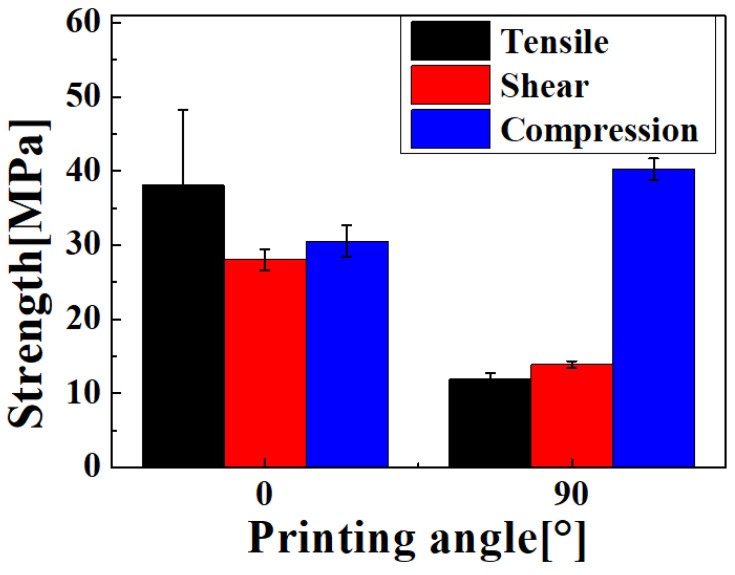
Breaking strength of each specimen according to printing angles.

**Figure 6:**
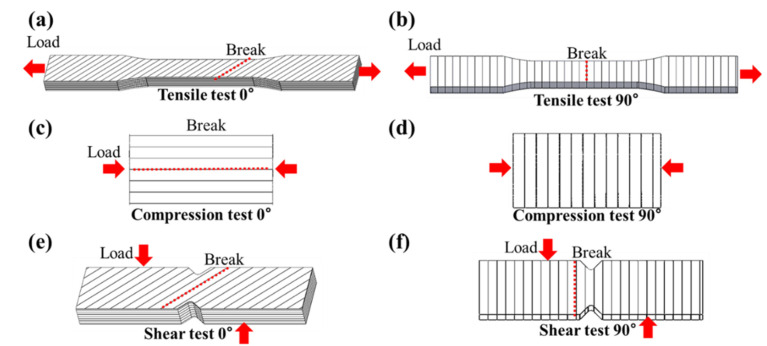
Concept figures for fracture planes of (**a**) 0° tensile, (**b**) 90° tensile, (**c**) 0° compression, (**d**) 90° compression, (**e**) 0° shear, and (**f**) 90° shear tests.

**Figure A1:**
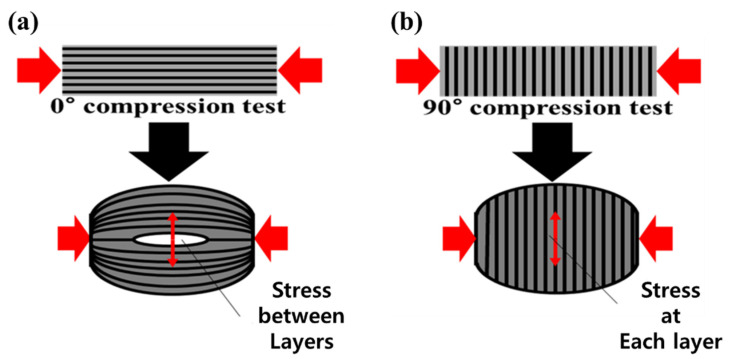
Schematic figure of fracture in the compression test according to the printing angle of (**a**) 0 deg and (**b**) 90 deg, respectively.

Figure 4 shows a picture of the fracture of the material test specimen for each condition. In the tensile test, if the printing angle was 0°, the average fracture strength and standard deviation were approximately 38.1 ± 10.13 MPa, which was approximately 3.2 times higher than that of the fracture strength at a 90° printing angle with 11.9 ± 0.78 MPa, as shown in Figure 5. In particular, there was a fracture between the stacked layers at 90°. In the case of the 0° printing angle, there was a fracture between the adjacent lines in the infill pattern at the same stacked layer, as shown in Figure 6. In other words, fracturing in the tensile test occurred between adjacent layers, and not at the PLA material itself. This phenomenon was the same in the shear test, as shown in Figure 4e,f. The fracture also occurred between the stacked layer and adjacent layer, and the average fracture strength and standard deviation were 28.1 ± 1.40 MPa at 90°, which was approximately 2.01 times larger than the 13.9 ± 0.46 MPa at 0°. Conversely, the compression test did not break the specimen, as shown in Figure 4c,d. The values of strength at 0° and 90° were −30.1 ± 2.11 MPa, and −40.26 ± 1.46 MPa, respectively. For the compression test of the 0-degree specimen, the stress vector that was applied to the specimen by the compressive load occurred between the stacked layers, as shown in Figure A1. On the other hand, for the 90-degree specimen, the stress vector occurred at each layer of the specimen. The specimen fabricated by the FDM method had a weak adhesion between stacked layers. Therefore, in the compression test, the compressive strength at 90 degrees was higher than at 0 degrees.

These results show that in the case of objects made by the FDM method, the low binding force between the stacked layers and adjacent layers has a more significant effect on the fracture strength than the rigidity of the PLA itself. This is an important finding that should be considered in the fabrication of capsules, where the isotropy of the fracture strength under the applied load should be guaranteed. Therefore, in capsule design, fabrication, and destruction experiments, tensile loads in the direction of the Z-axis and vertical loads parallel to the XY plane should be considered separately. Through these specimen experiments, the mechanical parameters of the FDM PLA specimen were obtained, as shown in Table A2. In particular, the values for the Poisson ratio entry of the FDM PLA specimen were first obtained, which was not studied in previous papers.

### 3.2. Finite Element Method Analysis of 3D-Printed Capsules

**Figure 7:**
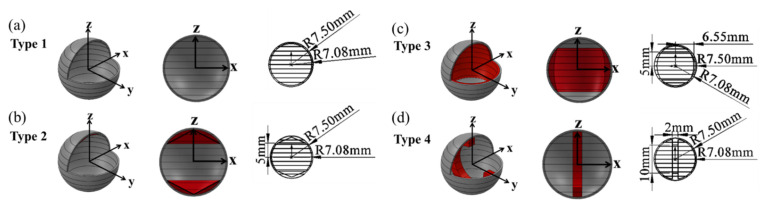
Four types of capsule design including detailed design parameters of (**a**) type 1, (**b**) type 2, (**c**) type 3, and (**d**) type 4.

**Figure 8:**
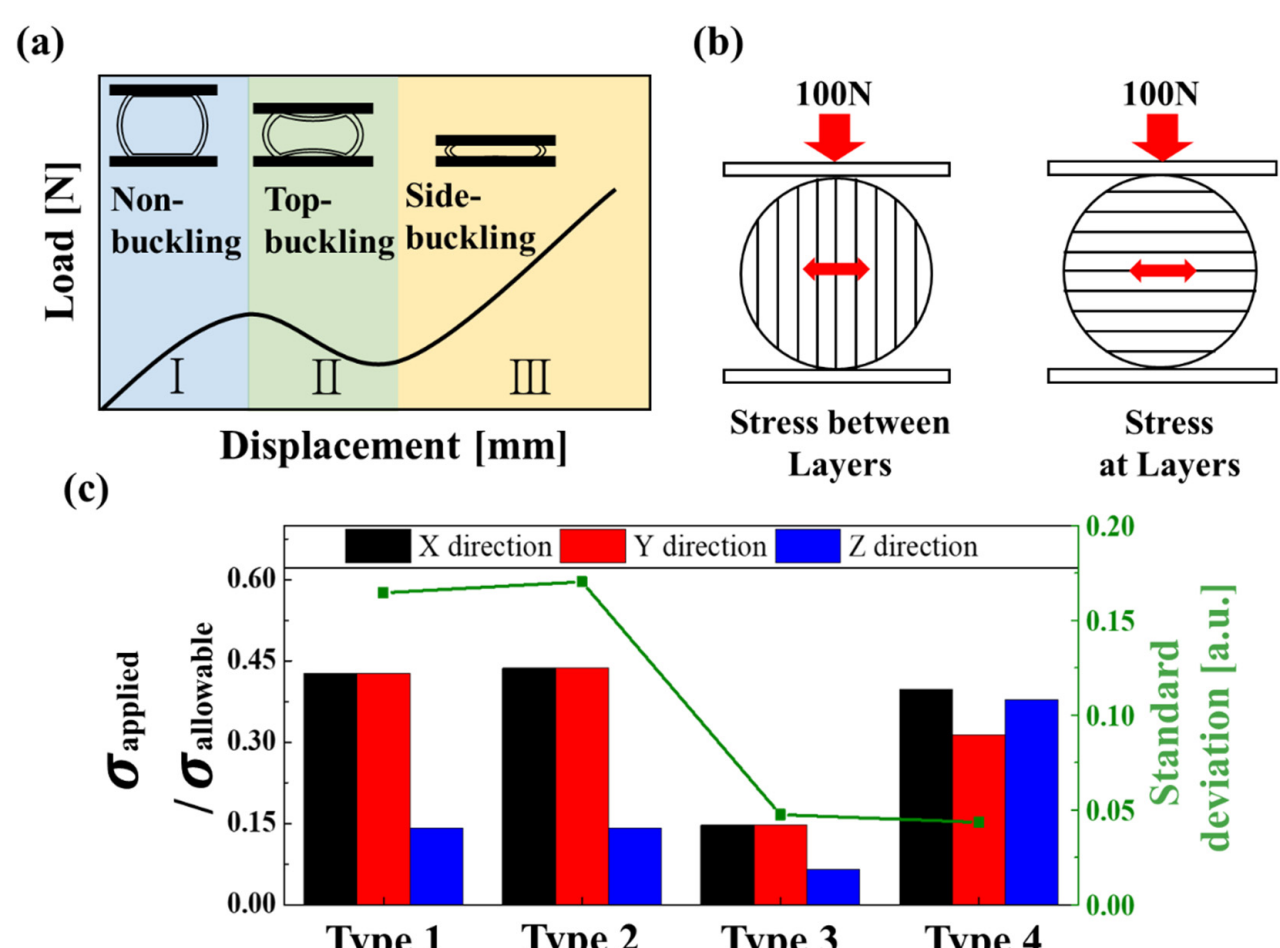
(**a**) General compression process of a hollow sphere. (**b**) Major stress factors according to applied load and printing angle. (**c**) ANSYS simulation results of the applied strength by allowed strength according to the direction of the applied load.

**Figure A2:**
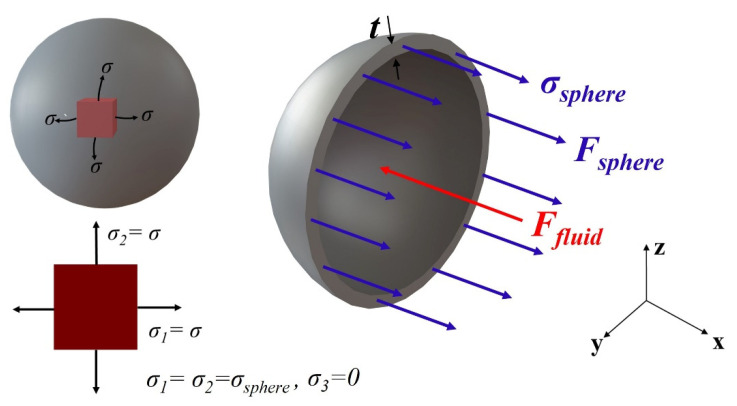
Spherical-capsule model and free body diagram for structural analysis.

Subsequently, FEM analysis was conducted, using newly measured mechanical parameters, with the ANSYS program (ANSYS inc, Daejeon, Korea, 2018 R2). Rather than immediately producing capsules for different types and conducting fracture experiments, the fracture strength for each proposed type was analyzed to design capsules with isotropic strength in all directions, as shown in Figure 7 and Figure 8 and Appendix A. The simulation parameters used in the FEM analysis were the same as in Table A3, and theoretical modeling of capsules containing self-healing solutions could be achieved mathematically using thin sheets of spheres, as shown in Figure A2 and Appendix A. Four types of capsules were designed, and Computer Aided Engineering (CAE) interpretations were performed on the fracture strength in accordance with the direction of their stacking and applied load in relation to the x-, y-, and z axes.

For each type shown in Figure 7, the z-direction is the direction in which the layer of the FDM PLA is stacked. Type 1 is a simple spherical capsule shape, and type 2 is a shape with enhanced thickness in the z-direction of the type 1 capsule, as shown in Figure 7. Type 3 is an enhanced shape of type 1 in the direction of the xy plane. Finally, the type 4 capsule is a structure with a ring reinforced in the z-direction, and is symmetric around the x-axis. The detailed design parameters for each type of capsule are shown in Figure 7.

Typical graphs of the applied load and capsule variations can be analyzed in three stages when compressing hollow spheres between plates, as shown in Figure 8a. Area 1 begins as soon as the hollow capsule and plate are in contact, and the load is applied. This is a section where the capsule can be elastically recovered just before buckling occurs.

In Area 2, buckling occurs at the part where the capsule and plate are in contact [36,37]. Area 3 is the section where the capsule is compressed after buckling while receiving a continuous load [48,49]. The strength of the capsule according to the direction of the compression force and stacked printing layers is generally measured and analyzed in Area 1 because the fracture occurs just before buckling. Therefore, it is determined that the corresponding load values where capsule fracture occurs should be similar in the x, y, and z directions to ensure an isotropic fracture strength.

**Figure A3:**
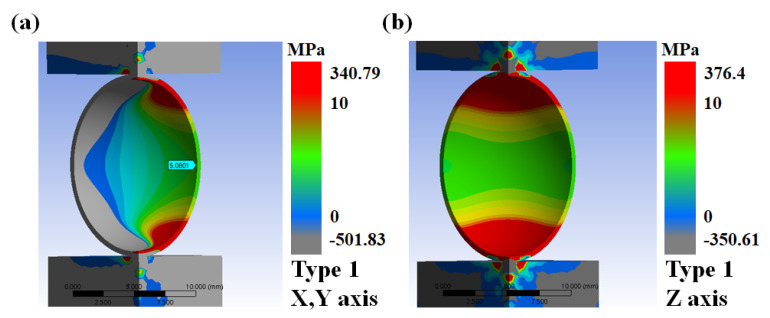
ANSYS simulation results: (**a**) type 1 x,y-axis direction compression, (**b**) type 1 z-axis direction compression.

**Figure A4:**
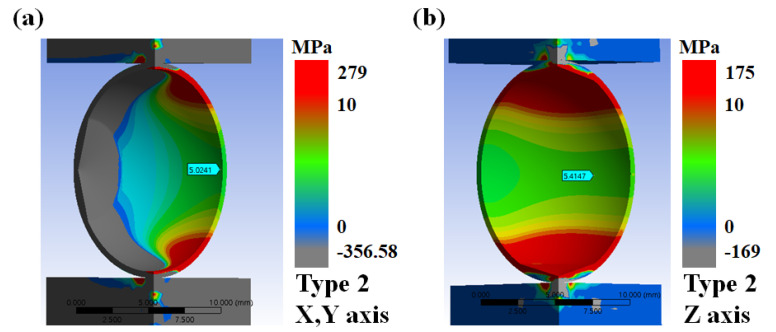
ANSYS simulation results: (**a**) type 2 x,y-axis direction compression, (**b**) type 2 z-axis direction compression.

**Figure A5:**
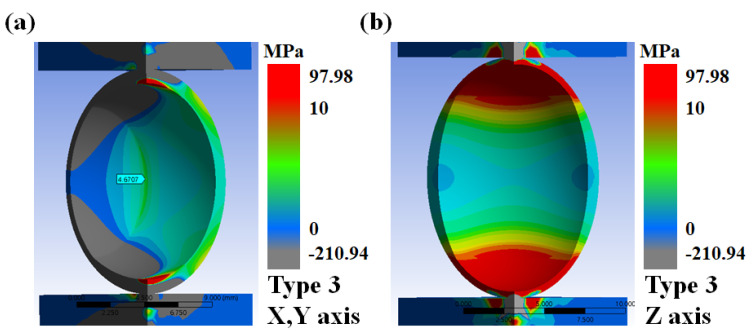
ANSYS simulation results: (**a**) type 3 x,y-axis direction compression, (**b**) type 3 z-axis direction compression.

**Figure A6:**
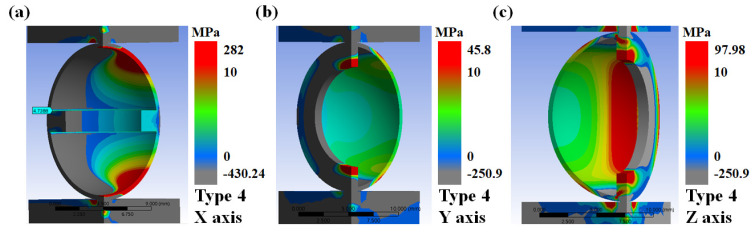
ANSYS simulation results: (**a**) type 4 x-axis direction compression, (**b**) type 4 y-axis direction compression, (**c**) type 4 z-axis direction compression.

Structural FEM analysis was performed on the four types of capsules, considering the aforementioned points, using the Ansys program. A 15 mm diameter capsule was constructed in a 0.2 mm mesh with a 20 (width) mm × 20 (depth) mm × 10 (height) mm size to perform a structural analysis of the compression load, as shown in Figure A3, Figure A4, Figure A5 and Figure A6. The ANSYS FEM analysis obtained the maximum tensile load at the equatorial center of the capsule where the fracture occurred for each capsule type. This equatorial center part of the capsule had the largest diameter and was a vulnerable area that was prone to breakage when a load was applied due to the low adhesion force between the stacked adjacent layers. In other words, as shown in the upper image of Figure 8b, tensile stress was applied between the adjacent layers when compressive forces were applied in the x and y axes. In addition, when compressive force was applied in the z-direction, tensile stress was applied, which caused the center layer to tear apart, as shown in the lower image of Figure 8b. These two factors should be considered in the FEM analysis as the main factors causing fractures.

To compare and analyze the degree of isotropicity of the capsule structure, 100 N was applied in the directions of the x-, y-, and z axes, respectively, for each capsule type, and the applicable strength of each material was derived through comparison with the allowable strength, as shown in Figure 8c. The type 1 capsule had the same value of 0.42 because it was symmetrical in the x and y axes, and had a value of 0.14 in the z-axis. The type 2 capsule also showed similar results, with 0.138 in the z-axis and 0.430 in the x and y axes, approximately three times larger. The reinforcement structure of type 2 had little effect on the fracture, and there was a large deviation in the x, y, and z directions. However, the type 3 capsule, designed to be thick in the xy-plane direction, had values of 0.142 in the x and y axes and 0.060 in the z-axis, showing a decrease in the difference between values for each direction. The type 4 capsule, with a ring reinforced in the z-direction, differed by up to 1.25 times, with values of 0.39 for x-axis compression, 0.31 for y-axis compression, and 0.37 for z-axis compression. Additionally, to analyze the isotropic strength in all directions, the standard deviation of the ratios of the applied stress to the allowable stress in the x, y, and z directions were compared, as shown in Figure 8c. Each capsule from type 1 to type 4 had values of 0.164, 0.170, 0.047, and 0.043, respectively. Based on these results, type 4 has the most uniform isotropic fracture strength for all directions.

### 3.3. Fracture Strength Tests of 3D-Printed Capsules

**Figure 9:**
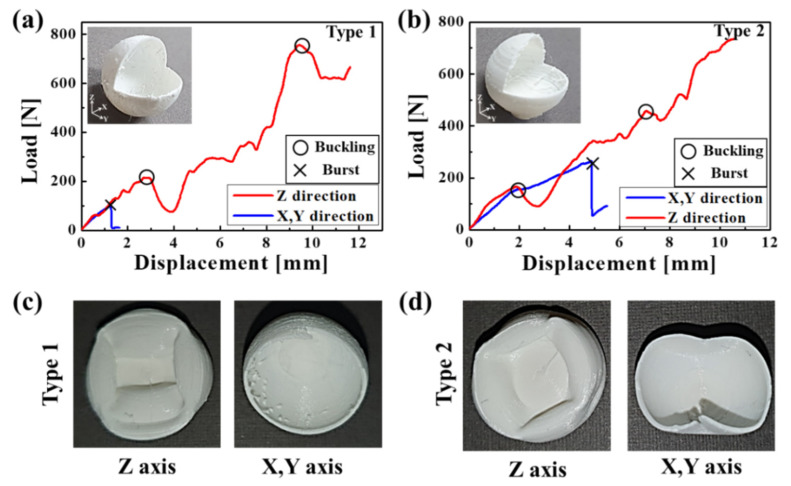
Graph for compressive displacement according to applied load for (**a**) type 1 and (**b**) type 2, and fractured capsule images of (**c**) type 1 and (**d**) type 2.

**Figure 10:**
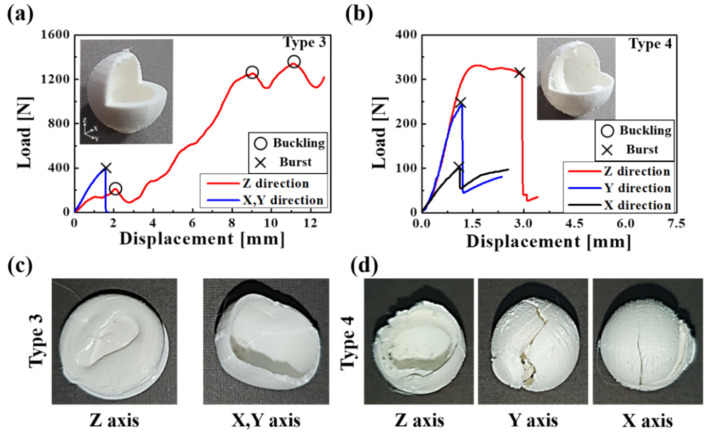
Graph for compressive displacement according to applied load for (**a**) type 3 and (**b**) type 4, and fractured capsule images of (**c**) type 3 and (**d**) type 4.

In addition, the actual capsules were produced using a 3D printer based on ANSYS interpretation, as shown in the inset images of Figure 9 and Figure 10. These capsules were printed to have a diameter of 15 mm at a nozzle of diameter 0.4 mm, layer height of 0.06 mm, and temperature of 210°, respectively. A compression test was conducted to verify the fracture strength of the manufactured capsules. The capsule was compressed at 10 mm/min, in accordance with the method of measuring the capsule strength used in previous studies [50,51,52]. In the case of type 1, the capsule burst before buckling when compressed at a load of 98 N, in the x and y directions, by approximately 1.27 mm, as shown in Figure 9a,c. However, in the case of z-axis compression, the first buckling occurred at 213 N at 2.87 mm compression, and the second buckling occurred at 755 N at 9.45 mm compression. In addition, the capsule was constantly compressed without bursting. Figure 9c shows capsules that were continuously compressed in the z-direction and burst in the x and y directions, respectively. Type 2 capsules, similar to type 1, showed buckling at 164.8 N when compressed by 1.85 mm, without bursting, and continued compression in the z-direction. In the case of x and y axis compression, the break occurred at 262.8 N before buckling when compressed by 4.87 mm. Type 3 showed continuous compression, buckling without the capsule bursting when compressed in the z-direction at 208.9 N, 1251 N, and 1338 N, respectively. Although it is ideal to have isotropic strength for capsules, types 1, 2, and 3 showed different results for each direction. In particular, bursting occurred easily in the x and y directions, while structural limitations in the z-direction resulted in continuous capsule compression with buckling and without bursting. In contrast, type 4 showed bursting occurring before buckling evenly in all directions of the x-, y-, and z axes, as shown in Figure 10. The break occurred at 102 N and 244 N due to compression of 1.11 mm and 1.19 mm in the x and y directions, respectively.

**Figure 11:**
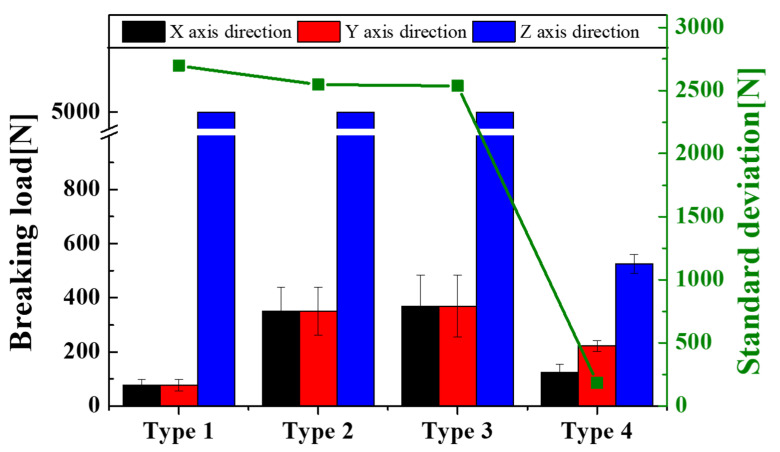
Capsule breaking load and standard deviation according to capsule type and direction of applied load.

Additionally, in the z-direction, the break occurred at 309 N due to compression at 2.93 mm. The explosion of type 4 capsules before buckling was attributed to the unique structure of the capsule with a ring reinforced in the z-direction, which was symmetric around the x-axis. Figure 11 shows the average burst load and the standard deviation of a capsule compressed three times in the x-, y-, and z axes of each type. Although each type of capsule should be burst in order for the healing solution located inside the capsule to flow well, type 1, 2 and 3 did not burst as a result of compression in the z-direction n even when compressed to the maximum compressive load of 5000 N of the equipment. Types 1, 2, and 3 were broken only due to x- and y-axis compression at 76.03 ± 21.25 N, 351.23 ± 88.5 N and 396.4 ± 113.87 N, respectively. Conversely, type 4 successfully burst in all directions of the x, y, and z axes at 123.3 ± 30.28 N, 221.3 ± 19.66 N, and 525 ± 35 N, respectively. The ring structure designed in type 4 is a key design parameter, which prevents buckling in the z-axis direction and induces the bursting of the capsule. To evaluate the isotropic properties of each capsule type, the standard deviation values for the burst load of the x-, y-, and z axes were calculated as shown in the y-axis to the right of Figure 11. As a result, the standard deviation of type 4 with bursting occurring in all directions was 183 N, which was approximately 14 times higher than the values of 2697 N, 2547 N, and 2537 N for the type 1, 2, and 3 capsules. These results show that the type 4 capsule is the capsule with the most isotropic fracture strength among the four types. However, this study has the limitation that the fracture strength test was conducted without healing solution in the capsule and this experiment was not carried out within the actual concrete environment.

## 4. Conclusions

In summary, a new self-healing capsule structure, based on 3D printing and using the FDM method, was proposed. The mechanical properties of FDM PLA were investigated through tensile, shear, and compression tests of the PLA specimens. Additionally, ANSYS FEM analysis was performed using these mechanical properties to design capsules with isotropic fracture strength. Based on the results of the analysis, four types of capsules were fabricated using the FDM 3D printing method; the characteristics of fracture strength were subsequently investigated through compression tests. By analyzing the fracture strength along the x, y, and z axes, it was verified that the type 4 capsule had approximately 14 times better isotropic strength properties, with a standard deviation value of 183 N for the burst load, than conventional capsule designs, which had a value 2697 N for type 1. However, in the future, it will be necessary to test the fracture experiment and the concrete healing process within the actual concrete using a designed capsule with isotropic fracture strength. The structural and analytic study of our proposed capsule will be valuable for improving the degree of isotropic fracture strength and accelerating 3D printing-based self-healing capsule research in the architectural engineering fields.

## Data Availability

Not applicable.

## Appendix A

**Table A1:** Composition and properties of PLA material.

PLA Chemical Structure	PLA Components	Properties	Values
	PolyLactic Acid (>90%)	Melt mass flow rate	6.09 g/10 min
	N,N’- Ethylene Bistearamide (<5%)	Melting temperature	145~160 °C
	Typical antioxidant (<5%)	Diameter	2.8 mm
	Typical pigment (<5%)	Printing temperature	190~240 °C

**Table A2:** Mechanical properties of PLA specimen printed FDM method.

Mechanical Properties		Values
Tensile strength	X-axis direction	38.11 (MPa)
Tensile strength	Y-axis direction	38.11 (MPa)
Tensile strength	Z-axis direction	11.9 (MPa)
Young’s modulus	X-axis direction	3.06 (GPa)
Young’s modulus	Y-axis direction	3.06 (GPa)
Young’s modulus	Z-axis direction	2.64 (GPa)
Shear strength	XY plane	13.89 (MPa)
Shear strength	YZ plane	28.05 (MPa)
Shear strength	XZ plane	28.05 (MPa)
Shear modulus	XY plane	0.981 (GPa)
Shear modulus	YZ plane	0.937 (GPa)
Shear modulus	XZ plane	0.937 (GPa)
Compression strength	X-axis direction	−30.05 (MPa)
Compression strength	Y-axis direction	−30.05 (MPa)
Compression strength	Z-axis direction	−40.26 (MPa)
Poisson ratio	XY plane	0.28 (a.u.)
Poisson ratio	YZ plane	0.33 (a.u.)
Poisson ratio	XZ plane	0.33 (a.u.)

**Table A3:** FEM simulation parameters.

Item	Properties	Value
Contacts	Type	Rough
	Update stiffness	Each iteration, Aggressive
	Pinball region	Radius
	Pinball radius	5 mm
Mesh(Triangular cell)	Physics preference	Mechanical
	Capsule element size	0.2 mm
	Plate element size	1.2 mm
	Type 1 nodes	316,944
	Type 1 elements	62,292
	Type 2 nodes	586,842
	Type 2 elements	360,104
	Type 3 nodes	778,166
	Type 3 elements	503,135
	Type 4 nodes	803,045
	Type 4 elements	515,354

Figure A2 shows the free body diagram of a spherical-capsule model for structural analysis. The model assumes a thin wall condition: the inner radius *r* to wall thickness *t* is greater than 10. Thus, the internal fluid force *F_fluid_* that acts horizontally against the plane is circular and can be expressed as follows:

(A1) $$F_{fluid}=P\cdot A=P\cdot \pi r^{2}$$

where *P* is the pressure, *A* is the plane dimension and *r* is the internal radius of the capsule. In addition, the horizontal force *F_sphere_* can be expressed as follows:

(A2) $$F_{sphere}=\sigma_{sphere}\cdot A=\sigma_{sphere}\cdot 2\pi rt$$

where *σ_sphere_* is the tensile stress, and *t* is the wall thickness of the capsule. Therefore, the equilibrium of forces in the horizontal direction can be expressed as follows:

(A3) $$\sum F=F_{sphere}-F_{fluid}=0$$

The normal stresses *σ_1_* and *σ_2_* are equal to the outer surface stress σsphere of the capsule and the *σ_3_* is zero. Thus, the obtained tensile stress is $\sigma_{1}=\sigma_{2}=\sigma_{sphere}=\frac{Pr}{2t}$. Considering the maximum shear stress, we should consider out of plane rotation about the x and y-axis. According to Mohr’s circle, the maximum shear stress can be expressed as follows:

(A4) $$\tau_{max}=\frac{\sigma_{max}-\sigma_{min}}{2}=\frac{\sigma_{1}-\sigma_{3}}{2}=\frac{Pr}{4t}$$
